# Identifying and characterizing social media communities: a socio-semantic network approach to altmetrics

**DOI:** 10.1007/s11192-021-04167-8

**Published:** 2021-10-12

**Authors:** Wenceslao Arroyo-Machado, Daniel Torres-Salinas, Nicolas Robinson-Garcia

**Affiliations:** EC3 Research Group, Department of Information and Communication Sciences, Faculty of Communication and Documentation, University of Granada, Granada, Spain

**Keywords:** Network analysis, Socio-semantic networks, Altmetrics, Twitter, Information science and library Science, Microbiology

## Abstract

Altmetric indicators allow exploring and profiling individuals who discuss and share scientific literature in social media. But it is still a challenge to identify and characterize communities based on the research topics in which they are interested as social and geographic proximity also influence interactions. This paper proposes a new method which profiles social media users based on their interest on research topics using altmetric data. Social media users are clustered based on the topics related to the research publications they share in social media. This allows removing linkages which respond to social or personal proximity and identifying disconnected users who may have similar research interests. We test this method for users tweeting publications from the fields of Information Science & Library Science, and Microbiology. We conclude by discussing the potential application of this method and how it can assist information professionals, policy managers and academics to understand and identify the main actors discussing research literature in social media.

## Introduction

Research literature is increasingly mentioned, shared and discussed on social media. This represents a substantial challenge as well as an opportunity to anyone trying to study the interactions that take place in the digital environment (Stieglitz et al., 2018). It provides researchers with major opportunities to develop novel methodological solutions by which to inform policy managers, journalists and information professionals on the way by which scientific literature is consumed. In vastly differing fields, many ad hoc solutions exemplify the growing interest in social media. In the field of science communication, for example, research has been conducted into the anti-vaccine movement on Twitter (van Schalkwyk et al., 2020), the dissemination of fake medical news (Waszak et al., 2018), or political communication and the influence of Twitter (Davis et al., 2017). In marketing, a substantial, growing number of social media metrics and analytics have been applied (Misirlis & Vlachopoulou, 2018). In disaster management, information propagated by social media such as Facebook and Twitter has formed the basis for new proposals (Kim & Hastak, 2018); and the digital humanities’ community on Twitter has been identified and analyzed (Grandjean, 2016).

In scientometrics, these studies have led to the emerging sub-field of altmetrics (Priem et al., 2010), in which mentions to scientific literature on social media are tracked to explore the social reception of research findings. However, this line of research has not been free of controversy. Initial high expectations of the potential value of tracking aspects of social or broader impact on research (Bornmann et al., 2019; Haustein, 2016) were soon rejected in the face of hard evidence (Robinson-Garcia et al., 2017; Sugimoto et al., 2017). Nonetheless, the relevance of social media in scholarly communication remains unquestioned (Robinson-Garcia et al., 2018; Wouters et al., 2019), leading to a new scenario in which novel metrics are being developed to understand and describe aspects of science communication that transcend traditional academic channels.

The rich variety of social platforms (Wikipedia, Mendeley, Twitter, and so on) has given rise to the development of altmetric data aggregators that provide data on a variety of social media sources. These include Altmetric.com, CrossRef Event Data, or Plum Analytics, among others. Despite the evident advantage of offering unique data access points, they do have limitations. Zahedi and Costas (2018) systemically compared altmetric data providers’ coverage, metrics and sources. They found differences in data collection, the identification and merging of different versions of a single publication, and data update periodicity. These can be added to other limitations directly related to the nature of social media and the concept of altmetrics, namely heterogeneity, quality and dependencies (Haustein, 2016).

For a variety of reasons, Twitter is the social media platform that has received most attention since the earliest days of altmetric studies. In part, this is because it is the public forum with the second-highest figures for coverage of scientific literature mentions after Mendeley (Robinson-Garcia et al., 2014). Nonetheless, while it is widely used by the general public, it has a relatively low level of acceptance among scientists. Most studies report that around 15% of academics have a Twitter account (Haustein, 2019), although the annual growth rate is constant (Joubert & Costas, 2019).

After initially promising results (Eysenbach, 2011), studies report that Twitter mentions to scientific papers poorly reflect citation impact (Haunschild & Bornmann, 2018). Furthermore, the inclusion of automated bots (Haustein et al., 2016) and the un-informative way in which scientific papers are tweeted (Robinson-Garcia et al., 2017) question the extent to which simple counts of tweets mentioning papers can be informative. Many studies have focused on characterizing the Twitter profiles of individuals who tweet scientific literature to better understand who they are (Díaz-Faes et al., 2019; Ke et al., 2017). The present study adds to this growing trend in the literature by proposing a methodological approach through which communities of actors can be identified on the basis of their scientific preferences. Our goal is to develop tools that can inform on targeted groups interested in specific topics which can later be characterized by other methods, as mentioned earlier. To achieve this, we build on previous studies that investigated differences in topics of interest across social media platforms (Arroyo-Machado et al., 2019; Robinson-Garcia et al., 2019).

The paper is organized as follows: first, we briefly review the literature and focus on three specific topics, Altmetric studies, studies specifically about Twitter, and studies relating to mapping and visualization techniques. Secondly, we formulate our objectives. We then describe our data retrieval and data processing and present our methodological proposal. We apply this in the field of Information Science & Library Science and in the field of Microbiology. We conclude by discussing our findings.

## Background

### Altmetric studies

Altmetrics were formally proposed in 2010 with the publication of the Altmetrics Manifesto (Priem et al., 2010), although similar proposals had appeared previously (Neylon & Wu, 2009; Nielsen, 2007; Taraborelli, 2008). The emergence of altmetrics led to a fundamental transformation of the field of scientometrics. This occurred at a time when different metrics, sources and indicators co-occurred, moving the field from an almost universal dependence on certain bibliometric databases to a heterogeneous range of data sources. Although scientometricians acknowledged the technical limitations of altmetrics from the very beginning (Torres-Salinas et al., 2013), an overall optimism led many to consider them an alternative to citation metrics and compared and analyzed their relationship with traditional metrics (Costas et al., 2015; Thelwall, 2018). But, apart from Mendeley (Thelwall, 2018), evidence only suggests the existence of a weak positive correlation.

This led to a change in the discourse and altmetrics began to be presented as a complement to citations (Haustein et al., 2015), rather than an alternative. While acknowledging their potential to inform on other indicators of scientific information consumption, there seems to be a consensus that they cannot be interpreted uniformly and that context plays an important role in their interpretation. This has led many to refer to altmetric indicators as metrics that capture an ‘unknown impact’ of scientific outputs (Bornmann et al., 2019; Kassab et al., 2020).

Since then, effort has been directed at studying the context in which this unknown impact is produced, identifying new channels of scholarly communication that go beyond the traditional (Holmberg et al., 2019). This shift has led some authors to refer to these new studies as studies on social media metrics (Wouters et al., 2019) and define them as ‘second generation metrics’ (Díaz-Faes et al., 2019). While the previous one transferred the citation model to social media, here the focus is on the activity and interactions that take place on social media. This leads to a new scenario in which the altmetric research is focused on the relational attributes of the social media activity rather than focusing on features (i.e., impact) related to scientific publications. To do so, the methodological framing has also changed, focusing now on techniques which help discover and analyze different kinds of social interactions (Costas et al., 2020) that allow a better understanding of science-society relations. However, these new approaches focus mainly on researchers discovering and topic visualizations in social media. But how can communities of social actors with the same interests be identified? Can communities of social actors who consume scientific literature outside the scientific realm be identified?

Numerous examples of these novel approaches to the use of altmetrics can be found in the literature. Table 1 summarizes 14 such methodological proposals. Essentially, these fall into three categories of application or approach: identification and characterization of researchers; visualization of topics discussed; and knowledge maps, which center on descriptive analyses and co-citation and co-word network analyses. Also, most of these studies revolve around the use of Twitter and Wikipedia. Colavizza (2020) estimated how well Wikipedia, as a tool communicating scientific knowledge to the general public, reflects current scientific progress on COVID-19. Similarly, science mapping techniques haven been used to analyze how Wikipedia structured science in comparison with global science maps based on bibliometric databases (Arroyo-Machado et al., 2020); and the humanities (Torres-Salinas et al., 2019).

**Table 1:** Main altmetric studies and methodological proposals by source of literature

	Application	Methodology	Source	Scope
Zahedi and van Eck (2018)	Profiling of Mendeley readers	Descriptive analysis and overlay visualizations	Mendeley	Multidisciplinary
Costas et al. (2017)	Identify researchers on Twitter	Rule-based methods	Twitter	Multidisciplinary
Ke et al. (2017)	Profiling of Twitter users	Descriptive and network analysis	Twitter	Multidisciplinary
Alperin et al. (2018)	Effectiveness of Twitter dissemination on outreach	Descriptive and network analysis	Twitter	Biomedicine
Robinson-Garcia et al. (2018)	Profiling researchers on Twitter	Social network analysis	Twitter	Multidisciplinary
Díaz-Faes et al. (2019)	Characterize Twitter communities interacting with science	Descriptive analysis and overlay visualizations	Twitter	Multidisciplinary
Haunschild et al. (2019)	Topic visualizations based on Twitter discussions	Co-word analysis	Twitter	Climate change
Hellsten and Leydesdorff (2019)	Topic and actor visualizations based on Twitter discussions	Co-occurrence of hashtags and mentions	Twitter	Biomedicine
Haunschild et al. (2020)	Topic visualizations based on Twitter discussions	Co-word analysis	Twitter	Library and Information Science
Robinson-Garcia et al. (2019)	Topic visualizations based on Twitter discussions	Social network analysis and overlay visualizations	Twitter	Microbiology
Torres-Salinas et al. (2019)	Mapping knowledge relationships in Wikipedia	Co-citation analysis	Wikipedia	Humanities
Arroyo-Machado et al. (2020)	Mapping knowledge relationships in Wikipedia	Co-citation analysis	Wikipedia	Multidisciplinary
Colavizza (2020)	Coverage of research topics in Wikipedia	Topic modeling and regression analysis	Wikipedia	COVID-19
Piccardi et al. (2020)	Measuring interactions with Wikipedia references	Engagement metrics	Wikipedia	Multidisciplinary

In addition to Wikipedia, other social media sources have also been used to study the dissemination of scientific activity. For instance, Mendeley has been studied to identify its user types’ interests in and their patterns of use of scientific publications (Zahedi & van Eck, 2018). However, in this respect, Twitter is the platform that has most frequently been studied.

### Twitter

Regarding the use of Twitter data, we find a first stream of studies that focus on identifying researchers or users who mention scientific publications and contextualize their activity. Among these we refer to studies like Ke et al. (2017), which identifies scientists from different disciplines; Robinson-Garcia et al. (2018), which proposes the use of mapping techniques to contextualize academics’ engagement in social media; or Díaz-Faes et al. (2019), which characterizes Twitter profiles mentioning scientific publications and identifies four dimensions of social media communication patterns.

Secondly, we find studies that focus on using Twitter activity to identify topics of interest. These studies attempt to explain differences between the way scientists communicate research and how research is perceived or characterized by Twitter users. They compare differences between Twitter hashtags and author keywords in tweeted publications (Haunschild et al., 2019, 2020); compare topics of interest by social media platform (Noyons, 2019; Robinson-Garcia et al., 2019); or associate instances of interaction and topic by comparing hashtags co-tweeted by the same profiles (Hellsten & Leydesdorff, 2019).

A third line of research is related to the diffusion of scientific publications. These studies aim to determine the social outreach attained by publications disseminated through Twitter (Alperin et al., 2018).

### Mapping and visualization techniques

One feature common to most of the aforementioned studies is their extensive use of mapping and visualization techniques. Based on network analysis, these techniques seek to construct n-dimensional spatial representations of science (Small, 1999). Most such representations are based on the co-occurrence of given events and are easily interpreted. From a bibliometric point of view, science maps are constructed from three elements: actors, resources and contents (Noyons, 2005), each of which offers a different level of analysis. In recent years, interest in mapping has grown as computational and methodological advances have extended their use. Furthermore, the number of visualization tools has increased considerably (cf. Cobo et al., 2011).

Originally, two types of co-occurrence links between similar publications were proposed: co-citation (Small, 1973) and bibliographic coupling (Kessler, 1963). Both were applied at different levels of aggregation (i.e., co-citation networks of authors [White & Griffith, 1981] or bibliographic coupling for journals [Small & Koenig, 1977]). But the number of co-occurrence types has grown to include co-author networks (Glänzel, 2001) or co-word maps (Callon et al., 1983), among others. Co-word maps facilitate the exploration of structures across the scientific landscape (Waltman & van Eck, 2012) as an alternative to citation networks (Boyack et al., 2005; Leydesdorff et al., 2013).

The emergence of new data sources and indicators, including but not exclusively from altmetrics, has led scientometricians to adapt these mapping techniques to the new metrics.

Hence, we find proposals to map scientific literature on the basis of the co-occurrence of publications downloaded by users (Torres-Salinas et al., 2014); to adapt the concepts of co-citation and bibliographic coupling to meet the context of the social media (Costas et al., 2020); and to create thematic landscapes by geographical region (Wouters et al., 2019). These methods can all be used in different contexts. For instance, Arroyo-Machado et al. (2020) created different levels of co-citation networks from Wikipedia entry references. Similarly, Haunschild et al. (2019) built thematic landscapes from co-tweets to visualize public discussion of specific research topics, while Díaz-Faes et al. (2019) used them to characterize the profiles of Twitter users who participate in scientific discussions on the social network. The co-use of hashtags in tweets mentioning scientific literature has also been proposed (Hellsten & Leydesdorff, 2019), as have follower-following networks of scientists who use Twitter (Robinson-Garcia et al., 2018). Clearly, scientific mapping techniques are being adapted to new environments and gaining complexity.

These techniques are based on the social network analysis of actors, relationships and structures (Wasserman & Faust, 1994). They represent any type of entity through nodes and establish relationships between entities that respond to co-occurrences, mentions, or any other type of interaction. Consequently, we can represent science-centered debates on social media at different levels and from different perspectives (Costas et al., 2020).

**Fig. 1:**
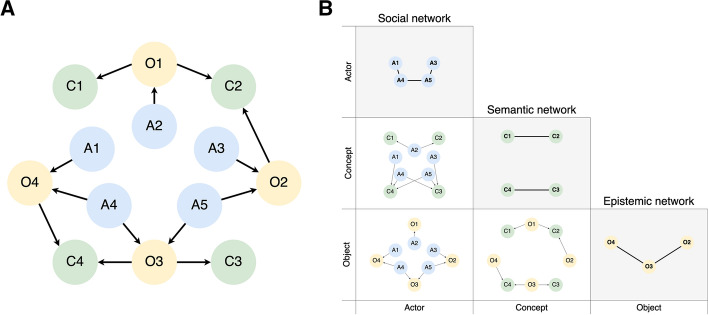
n-dimensional matrix constructed by combining the 2-mode matrices of objects, concepts and actors. This representation is based on the conceptual framework proposed by Hellsten et al. (2019)

The rationale behind social network analysis is that by combining co-occurring events, actors can be linked in a 2-mode (bipartite) network. Any such network is based on an asymmetrical matrix in which rows and columns are composed of different entities. Recently, Hellsten et al. (2019) suggested that by aggregating bipartite matrices different combinations could produce additional matrices. Figure 1a shows a 3-mode network that reflects differing but inter-related entities (actors, objects and concepts). Figure 1b shows how these matrices are constructed. Furthermore, the sub-matrices that appear in diagonal, show how entities of the same category are related through the combination of interactions between the other entities.

### Objectives

In the present paper we build on our literature review to better refine methods by which communities with common scientific interests can be identified on social media. We test our methodological proposal using Twitter mentions to scientific papers in two research fields: Information Science & Library Science and Microbiology.^1^ Our main objective is to present a methodological proposal based on social network analysis that allows us to identify cognitive communities by grouping actors who may not necessarily be socially connected but, rather, who are connected through their interests. A proposal that aims to contribute to the new generation of social media metrics (Wouters et al., 2019) as it allows to discover the implicit social and semantic relationships between actors based on the discussion around scientific publications through social media. To this end, we seek to achieve the following objectives:

1. To introduce a novel methodological proposal by which actors in a given network can be grouped on the basis of their cognitive interests thus, to some extent, removing social relationships that could potentially blur the boundaries between communities.
2. To test our methodical approach in a specific case study: Twitter mentions of scientific literature in the field of Information Science & Library Science.
3. To replicate this approach in a different field—Microbiology—to observe potential inconsistencies in the methodology and discuss differences between the two case studies.

Our study closely follows recent work in which a genuine effort has been made to conceptually define and then build a framework in which methodological solutions in the field of altmetrics can be expanded. For instance, Costas et al. (2020) recently proposed the concept of heterogeneous coupling in a study in which, from a theoretical perspective, they explored the potential of social network analysis to reveal links between the social media and science communication. Similarly, Hellsten et al. (2019) present their heterogeneous n-mode method which explores different combinations of interaction between actors. Our proposal could fit well into either of these two except for one noteworthy issue. The goal of our paper is to provide a practical application, showcasing a methodological innovation by which communities can be identified on the basis of common interests.

The present study builds on previous work which analyzed differences in interests of topic by social media platform (Robinson-Garcia et al., 2019) and by clusters (Arroyo-Machado et al., 2019). These earlier studies detected communities of actors who specifically mentioned the same publications and identified the topics that interested them.

## Data and methods

### Software

The data needed to reproduce our analyses are available at http://doi.org/10.5281/zenodo.4148941. We have included supplementary materials at https://doi.org/10.5281/zenodo.4332921. Network manipulation of co-word maps (semantic maps) was conducted using Gephi 0.9.2 visualization software (Bastian et al., 2009). As we want an easily replicable methodology fully based on social network analysis, the popular Louvain algorithm is used for community detection (Blondel et al., 2008). Social networks and the overlapping social and semantic networks were constructed using the igraph R package (Csárdi, 2020), and the Louvain algorithm was again used to detect social communities. Both social and semantic networks were tested with the Leiden algorithm (Traag et al., 2019) in Gephi and igraph. In both case studies, the results showed no significant improvements with respect to those derived from applying the Louvain algorithm, so we opted for the original version. Visualizations of intersection sets were constructed using UpSet R software (Lex et al., 2014), a visualization technique that defines the characteristics of the entities studied in order to group them. A detailed description of the data processing and the application of the entire process is available in an R Notebook at https://github.com/Wences91/social_media_communities. All methods have been automated and gathered under the R package ‘altanalysis’ (https://github.com/Wences91/altanalysis).

### Data gathering

We downloaded publication data for two research fields: Information Science & Library Science and Microbiology. We used the former as a case study to test our methodological approach. We then replicated the method in the latter field to compare results and analyze discrepancies in different contexts.

On 17 July 2019 we retrieved all records indexed in the Web of Science (WoS) InCites database (excluding the Emerging Sources Citation Index) published between 2012 and 2018 in the WoS categories of Information Science & Library Science (84,568 publications); and in Biotechnology and Applied Biochemistry (250,577 publications) and Microbiology (187,013 publications)—these two represent a combined total of 413,910 publications, henceforth referred to as ‘Microbology’. From Altmetric.com’s Altmetric Explorer portal, we extracted all social media mentions of these records by using their DOIs as our search item. Information Science & Library Science has 35,695 publications with DOI (42.21%), and Microbiology has 366,449 (88.53%). Table 2 summarizes the processing tasks undertaken prior to data analysis. We obtained the following datasets:

- Information Science & Library Science: 14,475 publications were mentioned by at least one altmetric source, giving a total of 167,110 mentions from Altmetric.com. Some 151,505 of these (90.66%) were Twitter mentions of 13,458 (92.97%) publications.
- Microbiology: 192,836 publications were mentioned by at least one altmetric source, giving 1,876,599 mentions from Altmetric.com. Some 1,599,315 of these (85.22%) were Twitter mentions of 173,406 (89.92%) publications.

**Table 2:** Summary of data processing of publications mentioned on social media in Information science and library science and microbiology

	Information science and library science				Microbiology			
	Publications	%	Twitter mentions	%	Publications	%	Twitter mentions	%
1. Download web of science’s in cites records from 2012 to 2018	84,568	100	–	–	413,910	100	–	–
2. Recover all Altmetric.com mentions to InCites publications	14,475	17.12	150,806	100	192,836	46.59	1,585,313	100
3. Data cleaning and filter mentions to only made from Twitter	13,446	15.9	150,723	99.94	173,306	41.87	1,579,896	99.66
4. Remove retweets	13,227	15.64	65,933	43.72	171,085	41.33	695,429	43.87
5. Retrieve web of Science author keywords and data cleaning	8452	9.99	35,336	23.43	101,206	24.45	327,449	20.66

Our purpose here is to map only those actors who are genuinely involved in Twitter discussions. Retweets have been excluded as they could potentially distort results: they correspond to the platform’s social function and do not necessarily indicate participation in scientific debate (Kassab et al., 2020). Twitter mentions retrieved via Altmetric Explorer do not distinguish between tweets and retweets. To identify retweets we searched the Twitter API between 26 December 2019 and 13 January 2020 and removed all retweets from our datasets. This cut the number of Twitter mentions in Information Science & Library Science to 65,933 (43.72% of the original dataset were individual tweets), and in Microbiology to 695,429 (43.87%).

Data processing enabled us to overcome specific limitations. Publications and mentions with no DOI or with a duplicate DOI, were excluded. We also extracted those user names that were missing from the original Altmetric.com dataset from the Twitter API. Thus, in Information Science & Library Science our dataset was further cut to 66,231 mentions (43.72% on Twitter) and in Microbiology to 699,507 (43.74%).

Simultaneously, we extracted author keywords of publications mentioned using terms included in the WoS Author Keywords. These are widely used in bibliometrics and have been previously applied in altmetrics (Haunschild et al., 2019, 2020). Furthermore, we conducted the following processing tasks. All records drawn from the Qualitative Health Research Journal (743 papers) were excluded since it would seem to have been misclassified because most citing journals belong to different categories (Supplementary material, Table C1). Including this journal distorts the semantic map (Supplementary material, Figure C1). Not all publications include author keywords and some journals are left out of the analysis. In Information Science & Library Science there are a total of 239 publication sources, and only 7 journals in the area with more than 10 publications do not include author keywords. From the 747 publication sources of Microbiology there are 18 journals in the area with more than 10 publications not including them either.

Our final Information Science & Library Science dataset constituted 8452 publications (63.9% of the total) with 44,421 keywords, of which 20,027 are unique, and 35,411 Twitter mentions (53.47% of the total); and in Microbiology, our final dataset constituted 101,206 publications (59.16%) with 540,227 keywords, of which 163,674 are unique, and 328,110 Twitter mentions (49.91%).

### Methodological proposal

We now describe our methodological proposal to identify communities of interest. This approach can be divided into three distinct phases.

Firstly, we construct a co-word network (semantic map) from the author keywords of publications tweeted in the field. The network is constructed regardless of the number of mentions received and is solely based on the co-occurrence of keywords in scientific publications. It is pruned to remove the weakest co-occurrences, less frequent keywords, and isolated components. Due to the different network sizes and edges’ weights (number of times than two keywords co-occur) in the two areas, the established minimums are not the same for both. This map enables us to identify research areas in the field. To do so, we use a social network community detection method. The chosen is the Louvain community detection algorithm (Blondel et al., 2008), where the quality function is the modularity value (Q). We seek a balance between the number and relevance of communities detected and the resulting modularity by applying different resolution values, a parameter which affects the size and number of detected communities. The minimum modularity value set to validate these communities is 0.3 (Newman, 2004). Then the detected communities are tagged taking into account an expert opinion.

Secondly, we assign social actors to topics identified in the map on the basis of the keywords in the papers they discuss. Mentions are combined with the keywords and clusters associated with the papers mentioned. This means that all mentions are divided into as many keyword groups as each paper contains.

**Fig. 2:**
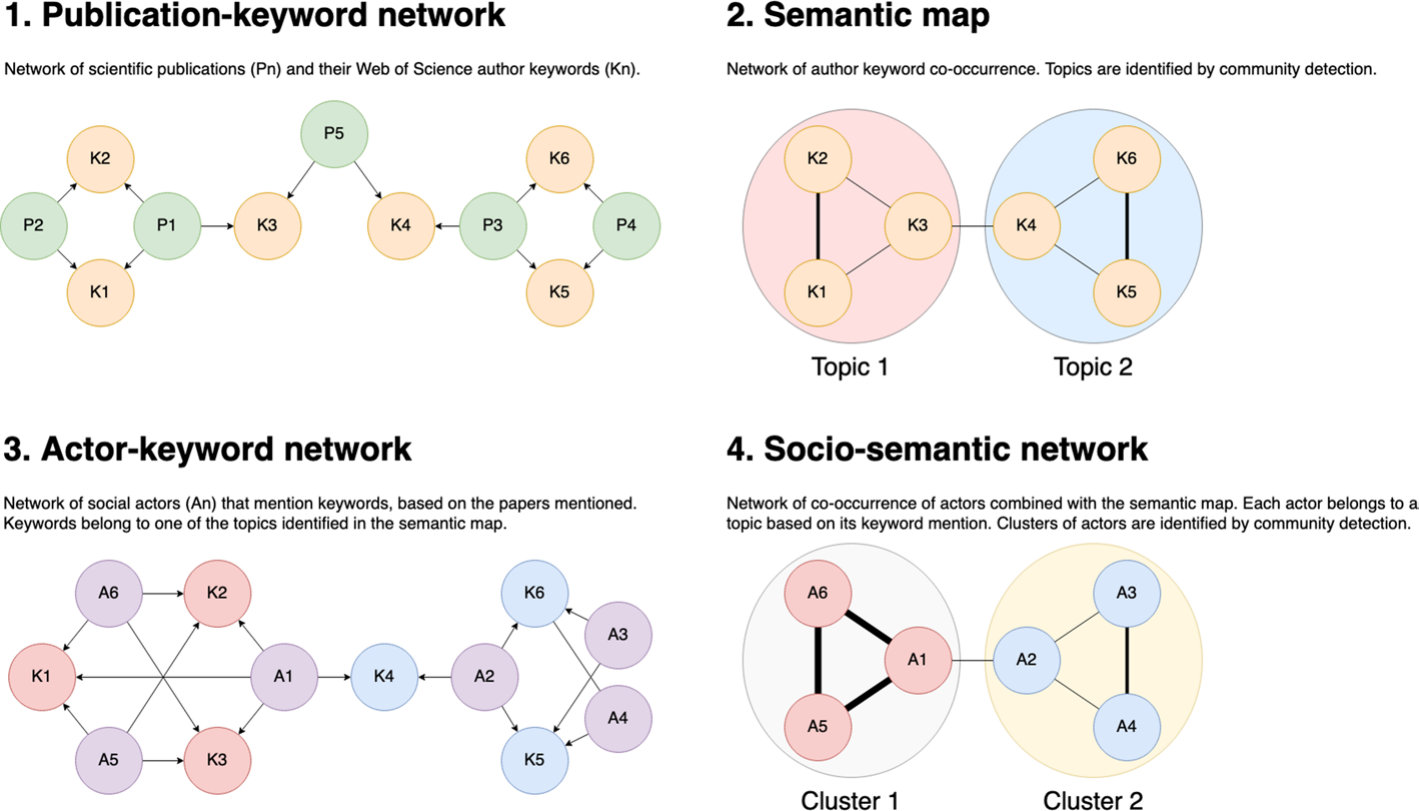
Overview of our methodological approach to identifying socio-semantic networks of Twitter users on the basis of commonly cited publications

Finally, we generate a network of social actors who are linked by the number of tweeted keywords they share (social network). This network is also pruned to remove the weakest relations also following a heuristic strategy, which means that there is no a standard value, but different tests are carried out for this purpose, and reduced to its main component. A community detection is applied to it, using the Louvain community detection algorithm and following the same criteria as in the semantic map. The resulting communities are reflected by areas. To generate the socio-semantic network, each social actor is assigned to its topic, generating a second grouping of social actors, whose quality is calculated by the modularity value. Figure 2 summarizes our approach.

## Case study: information science and library science

We identified a total of 13,243 Twitter users mentioning 8452 scientific publications of which 92.65% were articles and 3.42% reviews. Twitter users mention a mean 2.23 publications (SD ± 8.79) and 10.59 keywords (SD ± 32.32).

**Fig. 3:**
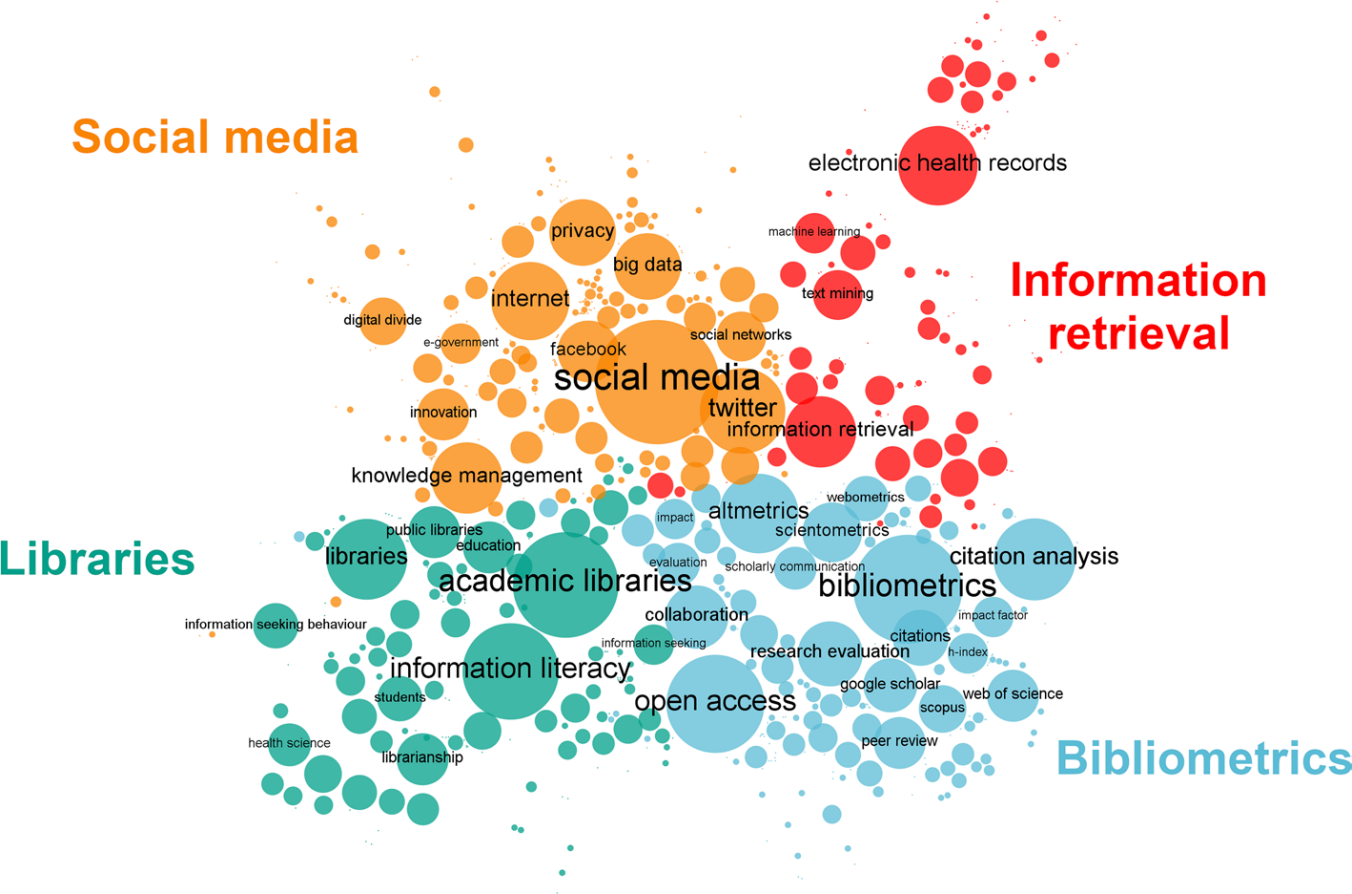
Information Science and Library Science thematic landscape. This map shows the main components of the network and those terms that co-occur 3 times or more. It contains 659 WoS author keywords

The author keywords co-occurrence network is composed of 20,025 nodes and 100,604 edges. It is reduced to 659 nodes and 1315 edges by removing edges with less than 3 co-occurrences and getting its main component. Figure 3 shows the resulting co-word map. We identified four clusters or topics by using a resolution value of 2.5 (Q = 0.62). These were tagged manually on the basis of expert opinions. We found these topics centered on social media (34.14% of nodes in the network), bibliometrics (26.56%), libraries (21.4%), and information retrieval (17.9%). The contents of the clusters were:

- Social Media: a community consisting of 5511 Twitter accounts, disseminating 2870 publications in 11,684 tweets and sharing 225 keywords. It includes publications related to social media use, the ethics of their use, their use by young people, and the application of big data techniques in social media analysis.
- Bibliometrics: a community consisting of 4989 Twitter accounts, disseminating 2229 publications in 11,984 tweets and sharing 175 keywords. This community includes publications related to bibliometrics and altmetrics analysis and covers issues relating to open science and science policy.
- Libraries: a community consisting of 2854 Twitter accounts, disseminating 1658 publications in 6297 tweets and sharing 141 keywords. This community includes publications relating to general, academic or specialized libraries, their evaluation, and the analysis and training of users.
- Information retrieval: a community consisting of 3522 Twitter accounts, disseminating 1486 publications in 7651 tweets and sharing 118 keywords. This community includes publications relating to information storage and retrieval, its application in electronic health records, the use of ontologies and classification systems and their interoperability.

**Fig. 4:**
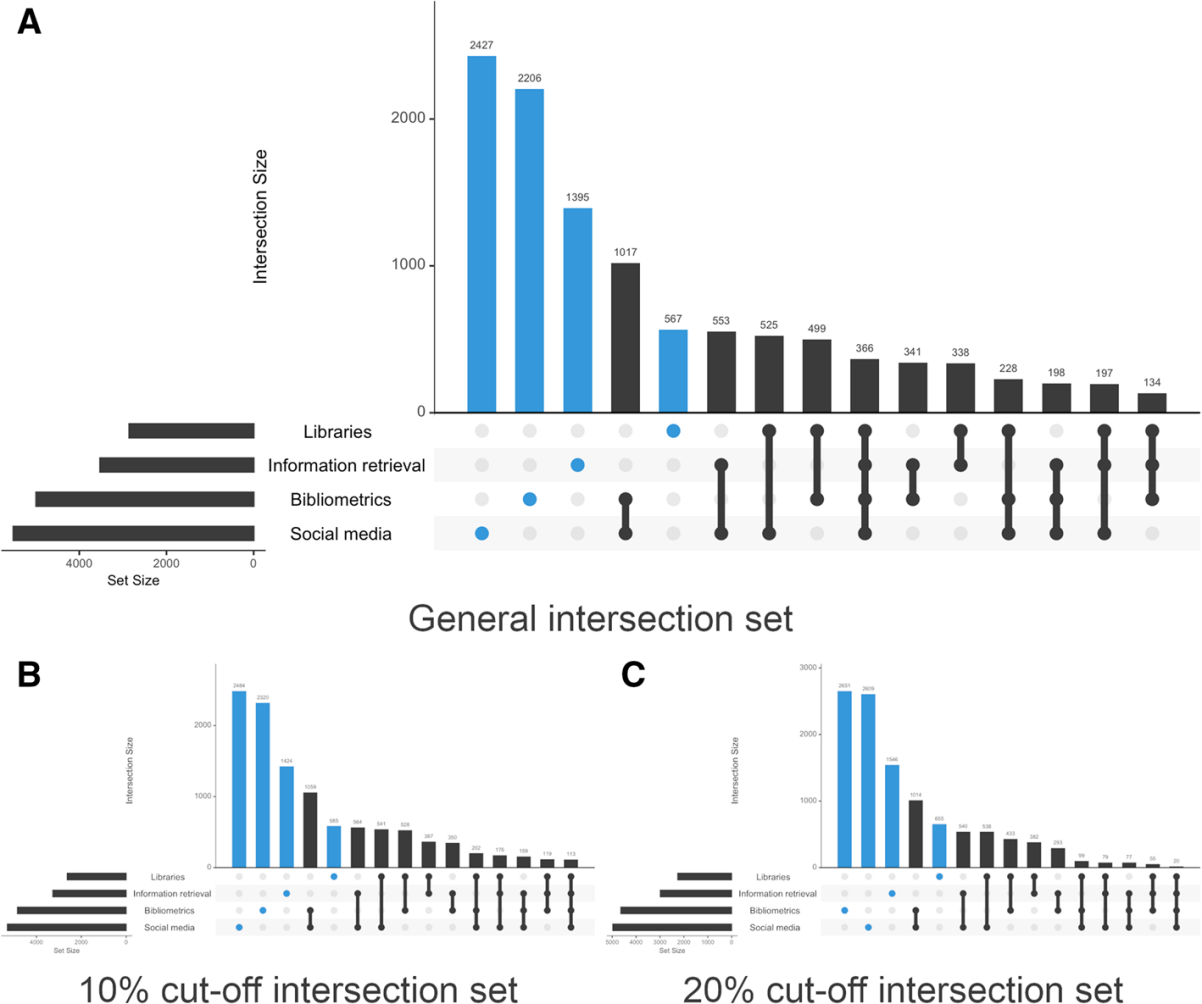
Intersecting sets for Information Science & Library Science. **a** corresponds to all combinations of actors and topics. **b** shows intersections after introducing a 10% cut-off for the number of times a keyword is mentioned. **C** shows intersections with a 20% cut-off point

Figure 4 shows the number of Twitter users associated with each topic. As we said earlier, while the largest groups constitute users who discuss topics related to a single area, we found many users who discuss topics related to different areas within the field. We identified 15 communities of interest: four consist of users clearly interested in a single area, whereas the rest combine interests from different areas. In our sample, 10,991 Twitter users (83%) mention one or more of the keywords from the four clusters detected in the semantic network. Those who mention keywords from a single community stand out: 2427 Twitter users discuss topics relating to commercial media (22.08%), 2206 bibliometrics (20.07%) and 1395 information retrieval (12.69%). Among those who refer to topics related to libraries, only 567 Twitter users (5.16%) exclusively mention keywords from this area.

Some 1107 Twitter users combine mentions to topics related with social media and bibliometrics (9.25%). In fact, 44.22% of those who discuss topics related to bibliometrics also discuss topics related to social media. This figure falls slightly when combined with information retrieval (39.61%) and drops further when combined with libraries (19.87%). Finally, one singular cluster is that consisting of 366 actors (3.35%) who mention all four topics.

**Fig. 5:**
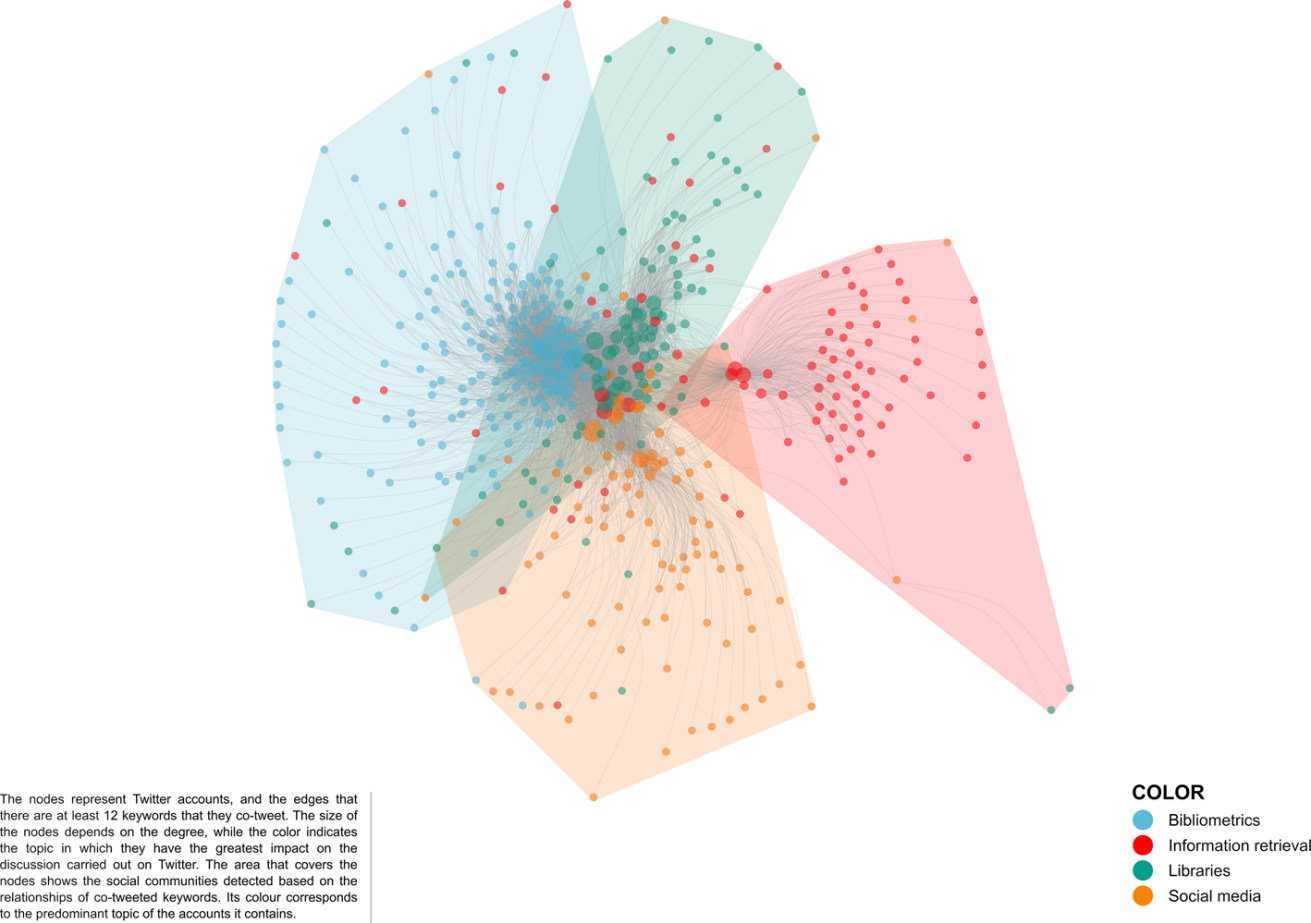
Information Science and Library Science socio-semantic network. Nodes are color-coded to identify the topics that have greater incidence. Edges are established on the basis of co-tweeted keywords. These have been filtered to a minimum of 12, and the corresponding communities are represented by overlapping areas

Figure 5 compares communities defined by co-tweeted keywords with those defined by co-occurring keywords in papers. Nodes represent Twitter users. They are colored-coded to reflect communities constructed on the basis of the co-occurring keywords (Q = 0.27). Areas are colored-coded to identify Twitter user communities constructed on the basis of co-tweeted keywords (Q = 0.32). As we have said, 96.69% of Twitter users tweeting keywords related to bibliometrics, form clearly-defined groups within this community regardless of the cut-off point applied (Fig. 4b, c). Similarly, 86.96% of users discussing keywords related to social media are grouped together regardless of the cut-off point applied. This percentage is lower in the case of users discussing topics related to information retrieval (64.29%) or libraries (61.54%). These results corroborate those of the profiles, in which users mentioning retrieval information and, especially, libraries who tend to show interest in a range of topics.

**Fig. 6:**
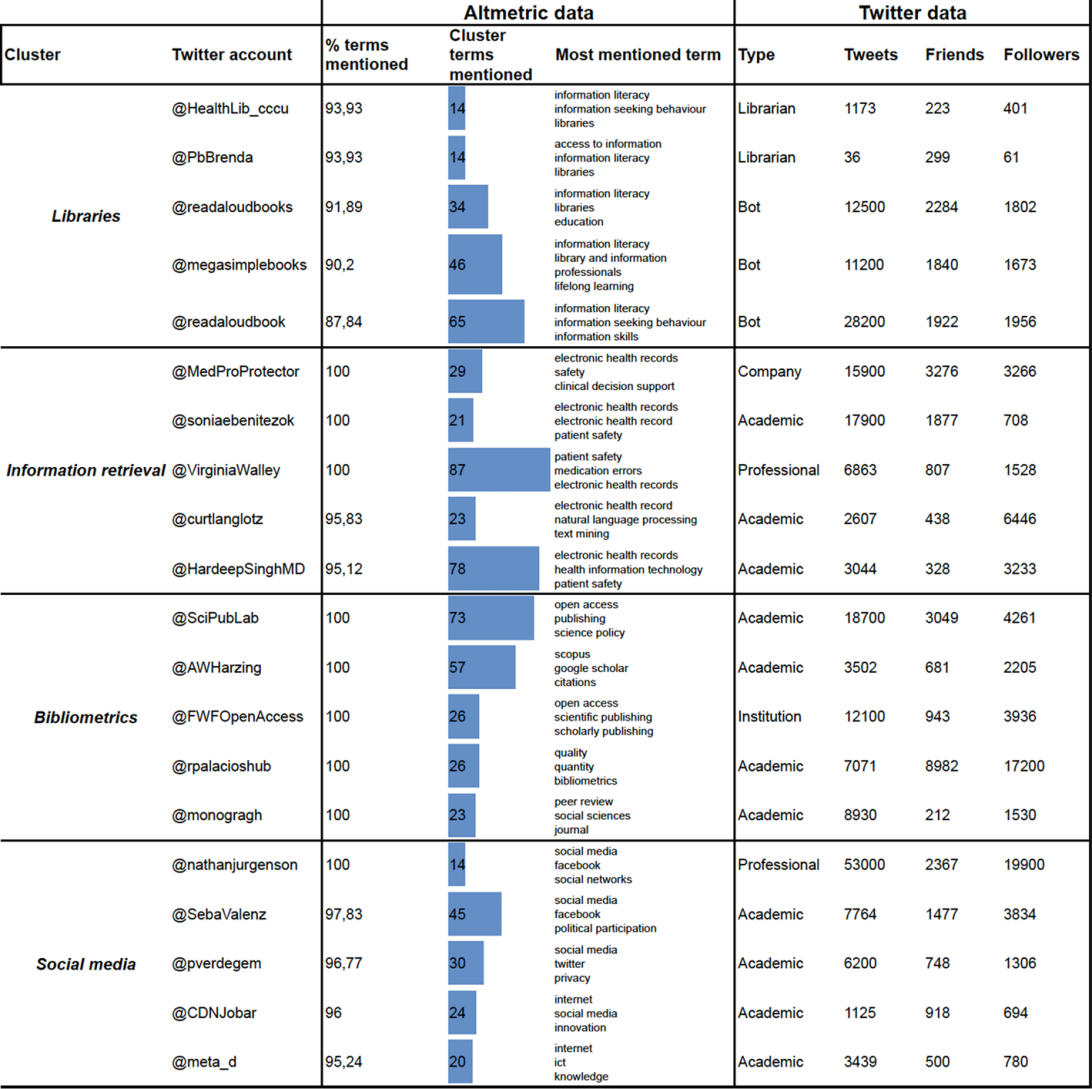
5 Twitter accounts with the highest percentage of terms mentioned for each topic

Figure 6 details the users belonging to each community and lists those with the highest percentage of terms in each area. We manually assign an account type to these 20 cases. While most of these users only focus on the area to which they have been assigned, we have found some broader profiles. We have also noted that, on the basis of the number of times keywords appear and the percentage of keywords mentioned, the most frequent users in the information retrieval and bibliometrics clusters are more active and engage more intensely with the topics related to their cluster. Finally, most of these users are academics although in the libraries cluster two accounts belong to librarians and three are bots.

## Case study: microbiology

We replicated our approach in a larger field—Microbiology—to see how it would work in a different context. We identified 48,109 Twitter users mentioning 101,206 scientific publications of which 86.52% were articles, 11.03% reviews, and 1.88% editorial material. Twitter users mentioned a mean 5.93 publications (SD ± 63.65) and 25.27 keywords (SD ± 197.84).

**Fig. 7:**
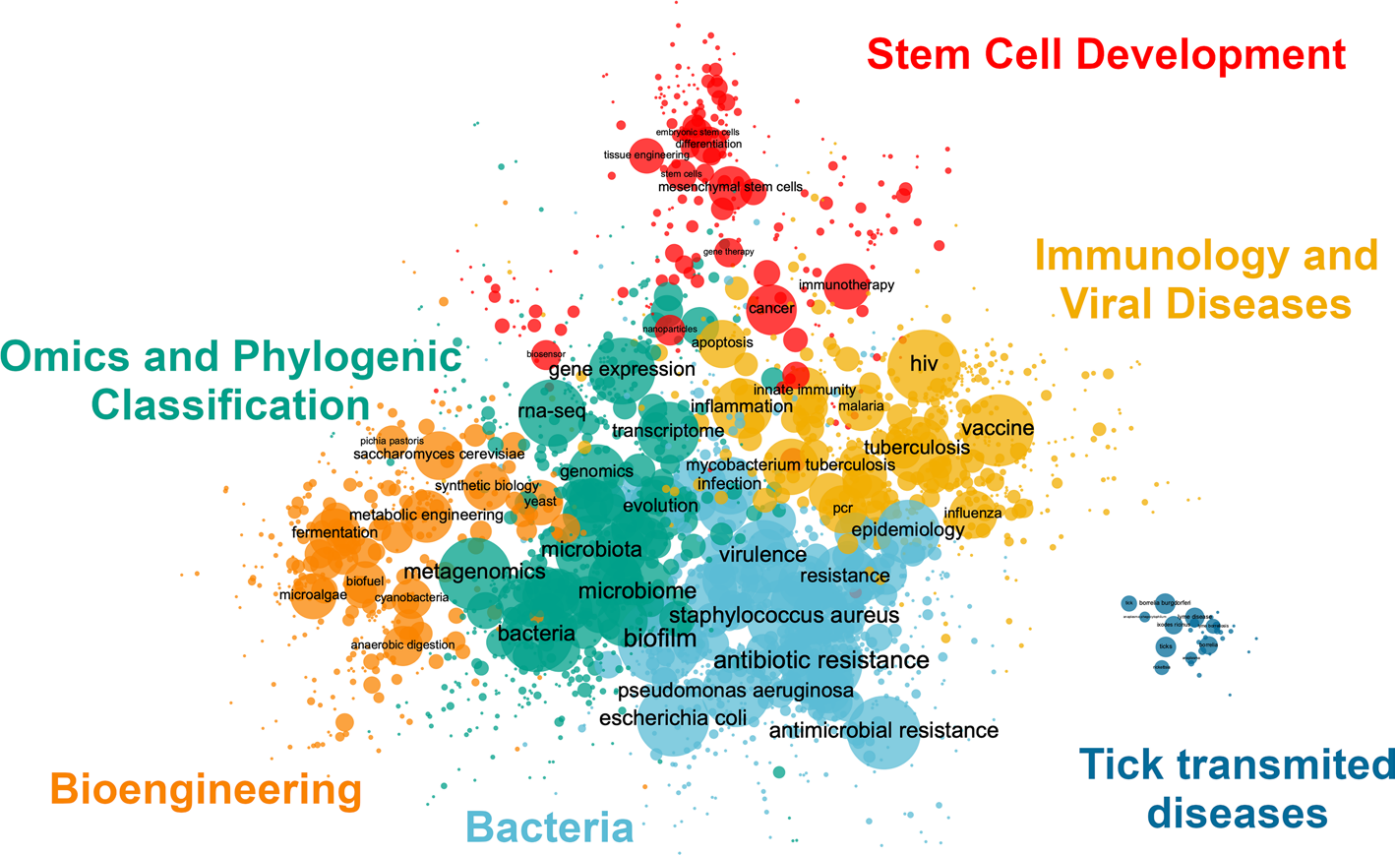
Microbiology thematic landscape. This map shows the main component of the network and those terms that co-occur 5 times or more. It shows a total 2309 WoS author keywords

The author keywords co-occurrence network is composed of 163,650 nodes and 1,173,938 edges. It is reduced to 2309 nodes and 7559 edges by removing keywords with less than 50 occurrences, edges with less than 5 co-occurrences and getting its main component. Figure 7 shows the corresponding co-word map. The community detection algorithm identified 6 clusters or topics using a resolution value of 2.0 (Q = 0.591). We labeled these: bacteria (28.58%); omics and phylogenic classification (25.6%); immunology and viral diseases (21.22%); bioengineering (13.64%); stem cell development (9.66%); and tick transmitted diseases (1.3%). The clusters’ contents were:

- Omics and Phylogenic Classification: a community consisting of 26,654 Twitter accounts, disseminating 35,450 publications through 143,604 tweets and sharing 591 keywords. It included publications covering studies of genetic material, bacterial microorganisms and biodiversity.
- Immunology and Viral Diseases: a community consisting of 18,695 Twitter accounts, disseminating 23,499 publications through 85,030 tweets and sharing 490 keywords. It is related to viral diseases, their diagnosis, novel treatments, and vaccines.
- Bioengineering: a community consisting of 11,523 Twitter accounts, disseminating 17,625 publications through 47,743 tweets and sharing keywords. It includes publications in biotechnology, metabolic engineering, and synthetic biology.
- Bacteria: a community consisting of 19,077 Twitter accounts, disseminating 33,805 publications through 111,915 tweets and sharing 660 keywords. It includes publications related to diseases of bacterial origin, epidemiology, and outbreaks of infectious diseases.
- Stem Cell Development: a community consisting of 7206 Twitter accounts, disseminating 11,208 publications through 31,081 tweets and sharing 223 keywords. It includes publications on regenerative medicine, gene therapy, and cancer treatment.
- Tick transmitted diseases: a community consisting of 1048 Twitter accounts, disseminating 1044 publications through 4477 tweets and sharing 30 keywords. It includes publications relating to tick and flea transmitted diseases.

**Fig. 8:**
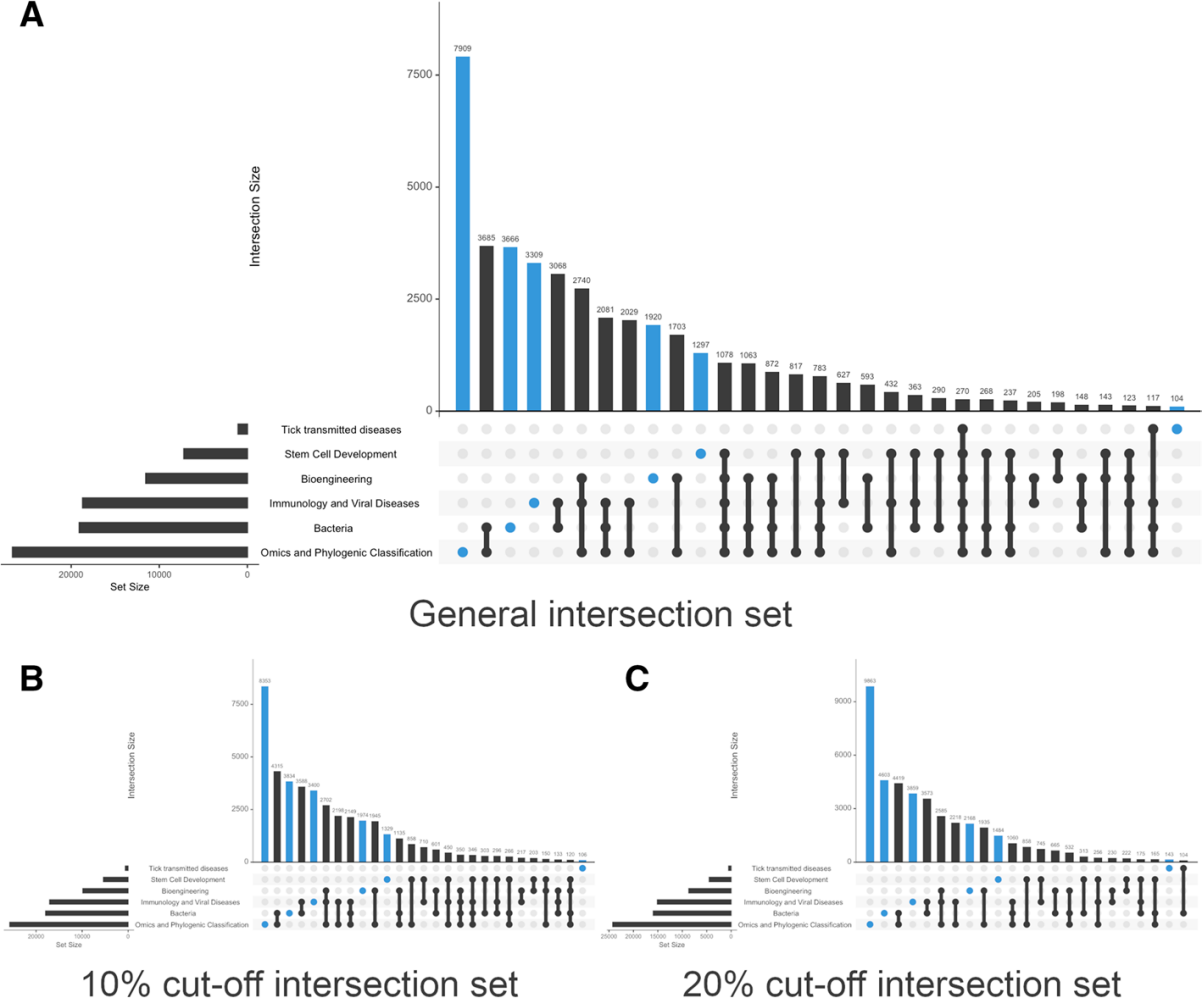
Intersecting sets with more than 100 actors in Microbiology. **A** corresponds to all combinations of actors and topics. **B** shows intersections after introducing a 10% cut-off for the number of times a keyword is mentioned. **C** shows intersections with a 20% cut-off point

When assigning Twitter users to each of these six topic groups (Fig. 8), we found a much more complex and varied picture than in the previous case study. We identified 58 communities of interest. Although Twitter user groups relating to a single topic still stand out (38.84% of all users), most groups show an interest in more than one topic. Some 7909 Twitter users only mentioned keywords relating to omics and phylogenic classifications (16.44%); 3666 mentioned keywords relating to bacteria (7.62%); 3309 immunology and viral diseases (6.88%); 1920 bioengineering (3.99%); 1297 stem cell development (2.7%); and 104 tick transmitted diseases (0.22%). The presence of ‘mixed’ profiles was much more common than in Information Science & Library Science. For instance, only 29.67% of Twitter users who mentioned keywords related to omics and phylogenic classifications solely discussed this topic. This fell to 19.22% in the case of bacteria, 18% for stem cell development; 17.7% for immunology and viral diseases; 16.66% for bioengineering; and 9.92% for tick transmitted diseases.

## Discussion

In the present study we propose a methodological approach to the identification of social media communities on the basis of common scientific interests. It enables us to link social media users on the basis of the keywords of the publications they mention and then group users by topic. We first applied this to Twitter users who mention publications in the fields of Information Science & Library Science. We then tested its feasibility by replicating the study in the field of Microbiology. Our proposal responds to the need for new efforts in social network analysis (Fu & Lai, 2020), is based on recently-published conceptual frameworks, especially the so-called heterogeneous couplings defined by Costas et al. (2020) and n-mode networks proposed by Hellsten et al. (2019), and previous studies in which we looked into differences in topics of interest on social platforms (Robinson-Garcia et al., 2019). This method is in line with the second generation of social media metrics (Díaz-Faes et al., 2019). Twitter mentions are not used here in a quantitative way, not even to filter keywords or actors. The focus of the paper is on social media-objects (Twitter users and tweets) and the papers are treated abstractly as keywords.

The resulting socio-semantic network of this proposal has significant differences with respect to other kinds of networks. 2-mode networks can reflect direct and explicit relationships, such as social actors mentioning publications, as well as implicit ones, such as social actors that are connected by co-mention of the same publications. All of them are easily readable, but when an n-mode network is constructed combining 2-mode networks it becomes complex to interpret. Not only do the nodes represent different kinds of entities, but the relationships that exist between them can be of a different nature. This hinders the analysis, especially when network pruning or community detection methods are applied. Our proposal is to overlap instead of adding 2-mode networks. In this way, communities are detected independently, and then joined. While the n-mode network communities are composed of different types of elements, for example social actors and keywords, in ours the social actors have two types of groupings, one based on their social relationships and the other on keywords mentioned by them. The overlap between the two allows determining if their social relations and interests are in line or differ.

Our study has not been free from limitations. Firstly, some tweets or accounts in our data sample were subsequently removed from Twitter or blocked. Consequently, they were excluded from our study. Second, to create the semantic maps, we initially extracted terms from publication titles. However, these proved too generic and included many distractors, generating widely varying communities. We resolved this by using WoS author keywords even though this limited the publications included to those present in the WoS database and having associated author keywords. Although actors were correctly assigned to the topic mentioned in most publications and people profiles prevail, bots are also present. In our Microbiology case study, given the complexity of the socio-semantic network, due to the variety of topics and social communities, this was not included.

Altmetrics has a number of well-known limitations—for example, the fact that data aggregators only retrieve tweets that include identifiers such as a DOI. The present study represents a step forward in the creation of applied solutions that use altmetrics beyond mere counting. Elsewhere, studies have already identified researchers (Costas et al., 2017; Ke et al., 2017) and communities on Twitter (Robinson-Garcia et al., 2018) or visualized the topics discussed on social media by using WoS author keywords and hashtags (Haunschild et al., 2019, 2020). Indeed, the thematic landscapes in this study seem more granular and more detailed than those generated elsewhere (Robinson-Garcia et al., 2019) due to our use of WoS author keywords instead of title noun phrases. Our study used both methods but integrates them into a single visualization. In this context, Hellsten et al. (2019) and Hellsten and Leydesdorff, (2019) proposed heterogeneous networks and applied these, respectively, to scientific journals and their attributes and Twitter and user mentions and hashtags. These proposals were based on networks produced by aggregating bipartite matrices that combine actors and objects in the same network. Our proposal also combines co-occurrence relationships of actors, publications and author keywords but we do not directly integrate them all into a network. Instead, we take the co-occurring keyword network and the co-tweeted keyword network and overlap these. Thus, the network is only formed of actors linked by social relations and their social communities are delimited through overlapping areas.

## Concluding remarks

Our proposed methodology allows us to identify communities of users in an inclusive way, reflecting a complex reality in which actors may be interested in different aspects of a research field. This is especially evident in the case of Microbiology, where there are many groups consisting of only a few individuals assigned to more than one area. This study furthers our understanding on the use of social media to inform on scientific literature consumption by the general public. By isolating communities of common interest as well as finding those with overlapping interest we can narrow the target audience who is discussing scientific literature in social media. This is potentially useful to assess on the effectiveness of social outreach of scientific research, identify social stakeholders or analyze communication strategies. Further research should consider combining methods such as the one proposed with those strictly focused on characterizing user types (cf. Díaz-Faes et al., 2019).

By focusing on concepts (i.e. keywords) rather than objects (i.e. publications), we minimize potential relationships derived from social relations between actors rather than from common research interests (e.g. colleagues from the same institution).

This methodology has the potential of being applied in other scenarios from the ones proposed here. Other social media platforms could be considered, as well as other types of contents shared through social media. Some of the many and varied contexts in which it can be applied are political participation and political engagement (Halpern et al., 2017), trolling interactions in the online gaming sphere (Cook et al., 2019), experiences of mental disorders shared in forums (Yoo et al., 2019), or social communities discussing eating disorders (Wang et al., 2017). Moreover, it is possible to use other social objects and links to construct the social network and other kinds of semantic maps, for example Reddit posts as social object, co-mentioned hashtags for social network, and topic modelling for semantic map. In the specific case of altmetrics, a future line of study is the application of this methodology to different social media and the use of other terms to create the semantic maps. This is an initial approach only using Twitter mentions due to their enormous coverage and the extension of altmetrics studies. However, we would hope to study its applicability further by using altmetric sources other than Twitter, to study source-related differences in the type of users who discuss scientific literature.

## Data Availability

All data are available at http://doi.org/10.5281/zenodo.4148941

## Footnotes

1 We selected two categories as distant as possible from each other. Information Science & Library Science and Microbiology belong to very different scientific areas (Social Sciences and Health Sciences) and have significant differences, both, in terms of volume of publications, and communication and collaboration patterns.

## Code availability

All the code with the data processing is available at https://github.com/Wences91/social_media_communities
